# Structure–Property Relationships in Transition Metal Dichalcogenide Bilayers under Biaxial Strains

**DOI:** 10.3390/nano11102639

**Published:** 2021-10-07

**Authors:** Pingping Jiang, Pascal Boulet, Marie-Christine Record

**Affiliations:** 1 Aix-Marseille University, UFR Sciences, CNRS, MADIREL, F-13013 Marseille, France; pingping.jiang@insa-rennes.fr (P.J.); pascal.boulet@univ-amu.fr (P.B.); 2 Aix-Marseille University, UFR Sciences, CNRS, IM2NP, F-13013 Marseille, France; 3 Univ Rennes, INSA Rennes, CNRS, Institut FOTON-UMR 6082, F-35000 Rennes, France

**Keywords:** two-dimensional materials, chalcogenides, photovoltaics, DFT calculations, QTAIM, structure–property relationship

## Abstract

This paper reports a Density Functional Theory (DFT) investigation of the electron density and optoelectronic properties of two-dimensional (2D) MX_2_ (M = Mo, W and X = S, Se, Te) subjected to biaxial strains. Upon strains ranging from −4% (compressive strain) to +4% (tensile strain), MX_2_ bilayers keep the same bandgap type but undergo a non-symmetrical evolution of bandgap energies and corresponding effective masses of charge carriers (m*). Despite a consistency regarding the electronic properties of Mo- and WX_2_ for a given X, the strain-induced bandgap shrinkage and m* lowering are strong enough to alter the strain-free sequence MTe_2_, MSe_2_, MS_2_, thus tailoring the photovoltaic properties, which are found to be direction dependent. Based on the quantum theory of atoms in molecules, the bond degree (BD) at the bond critical points was determined. Under strain, the X-X BD decreases linearly as X atomic number increases. However, the kinetic energy per electron G/ρ at the bond critical point is independent of strains with the lowest values for X = Te, which can be related to the highest polarizability evidenced from the dielectric properties. A cubic relationship between the absolute BD summation of M-X and X-X bonds and the static relative permittivity was observed. The dominant position of X-X bond participating in this cubic relationship in the absence of strain was substantially reinforced in the presence of strain, yielding the leading role of the X-X bond instead of the M-X one in the photovoltaic response of 2D MX_2_ material.

## 1. Introduction

Since the discovery and isolation of graphene [1], two-dimensional (2D) materials have tremendously attracted attention due to their unique physical properties. Among these materials, the transition metals dichalcogenides materials (MX_2_, with M = Mo, W and X = S, Se, Te) have shown to be interesting candidates for optoelectronic applications because they are stable, their layers bear no dangling bonds, and their bandgaps are ideally suited (see [2] and references therein). As an example, among the various exotic properties of MoS_2_ is the change from indirect to direct bandgap from the bulk or multilayered structures to the monolayered one [3,4,5]. More widely, the tunability of their structure and properties makes them suitable in, e.g., catalysis [6,7,8,9,10,11], energy storage and conversion [12,13,14,15,16,17], biomedicine [18,19,20,21,22,23], and sensors [24,25,26,27].

In a previous work [28], we investigated the layered-dependent structural, electronic, and optical properties of MX_2_ homo- and heterostructures by DFT calculations. The quantum theory of atoms in molecules [29] was used to process the electron density in order to correlate electronic interactions and macroscopic optical properties. We found that the static relative permittivity and the weighted bond degree summation are linked by a cubic relation and that the layered-dependent electronic and optical properties are mainly attributed to the interlayer X-X bonds. Furthermore, it has been reported in literature that the application of strains allows one to finely tune the transition metals dichalcogenides (TMD) electronic properties [30,31,32,33,34,35,36]. For instance, while uniaxially strained, the MoS_2_ monolayer changes from direct to indirect bandgap, which opens up the possibility of designing TMD through strain engineering. In addition, whereas conventional materials hardly bear strains exceeding a few percent, MoS_2_ has been shown to be able to withstand strains above 11% [37]. 

A great number of first principle calculations have already been reported on the effect of strain on the electronic properties of 2D semiconducting TMD [36,38,39,40,41,42]. However, these works almost all focus on monolayers. As for the effect of strain on the optical properties, it has only been investigated for monolayers. In a comparative investigation on MoS_2_ monolayers, Carrascoso et al. [43] showed that uniaxial strains have a weaker effect on the materials properties than biaxial ones. This type of comparative study has never been carried out on bilayer TMD, but we expect a similar trend. Hence, building upon our previous work, we investigated for the first time the effect of biaxial strains on both electronic and optical properties and the contribution of the interlayer van der Waals interactions in the optical properties in the bilayered MX_2_ compounds. 

## 2. Materials and Methods

DFT [44,45] calculations were carried out by a full-potential linear augmented plane wave method (FP-LAPW) as implemented in the program WIEN2k [46]. The generalized gradient approximation level of theory was applied with the Wu-Cohen (WC) functional [47]. During the optimization of the atomic position, the convergence criteria were set to 10^−5^ Ry and 1 mRy/Bohr for the energy and forces, respectively. The R_mt_K_max_ parameter was set to 7. Besides, the first Brillouin zone was sampled with 1500 k-points that were mesh selected according to the Monkhorst–Pack algorithm [48]. Although hybrid range-separated functionals are now recognized as a standard for obtaining an accurate description of chemical systems, the choice of the WC functional was made after systematic tests and comparisons with available data. It appears that this functional gives very reliable structural parameters compared to experimental ones. Regarding band structures, this functional also yields decent results, which may be attributed to the fact that we are investigating chalcogenides, for which the energy gap is rarely wrongly zeroed. In addition, considering the huge amount of calculations achieved in this work, we concluded that using a more elaborated functional, such as a hybrid one, would have led to such a computational cost that it could have jeopardized the achievement of our objectives.

**Figure 1:**
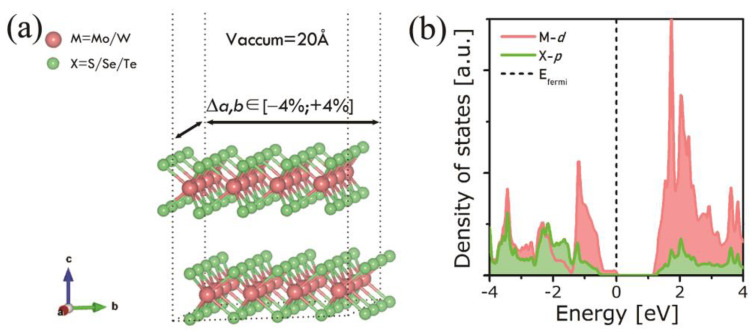
(**a**) Scheme of MX_2_ (M = Mo, W; X = S, Se, Te) bilayers used in this work. The applied strains are biaxial ones with ∆a (=∆b) ranging from −4% to +4%. (**b**) Calculated density of states of MoS_2_.

The investigated MX_2_ (M = Mo, W; X = S, Se, Te) bilayers consisting of 4 × 4 × 1 supercells and the density of states near the Fermi level (where MoS_2_ is taken as an example) are depicted in Figure 1. A 20 Å vacuum thickness was added atop to separate free surfaces, hence avoiding interaction between periodic images. The structures were then relaxed. Subsequently, both compressive and tensile in-plane biaxial strains were applied with values ranging from –4% to +4% by steps of 2%. Negative deformations stand for compressive strains, whereas positive ones stand for tensile strains. After structure relaxation, electronic band structures and optical properties (relative permittivity, absorption coefficient, extinction coefficient, and refractive index) were calculated for each of the six MX_2_ bilayers. The application of a biaxial strain on chalcogenide bilayers aims at reproducing the epitaxial strain on the absorber layer in real devices. Nonetheless, our model is limited by the absence of a substrate, which does not allow us to investigate the band alignments in the device. Hence, the energy discontinuities at the band edges that serve as the basis for controlling transport properties were not characterized.

Further, the electron densities obtained from WIEN2k calculations were processed with the Critic2 package [49], which implements the quantum theory of atoms in molecules [29,50], from which the total, kinetic, and potential energy densities at the bond critical points (BCPs) were obtained. The bond degree at each BCP was then calculated from the total energy density and the electron density [51].

## 3. Results

### 3.1. Electronic and Optical Properties

**Figure 2:**
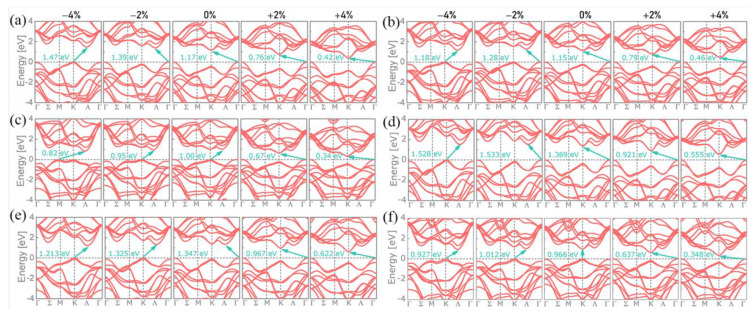
Band structures of (**a**) MoS_2_, (**b**) MoSe_2_, (**c**) MoTe_2_, (**d**) WS_2_, (**e**) WSe_2_, and (**f**) WTe_2_. For each panel, from left to right: −4%, −2%, 0%, +2%, and +4% applied biaxial strain.

**Figure 3:**
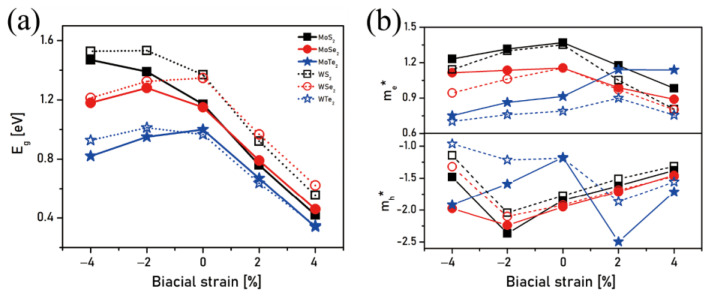
(**a**) Bandgap energies and (**b**) electron and hole effective masses (m_e_* and m_h_*, respectively) at the respective CBMs and VBMs with respect to applied strains from −4% to +4% for the MX_2_ bilayer structures (M = Mo, W; X = S, Se, Te).

The band structures of the six investigated MX_2_ bilayers under biaxial strains (from compressive to tensile ones) are depicted in Figure 2. For the unstrained bilayers (null strain), except for WTe_2_, all the bilayers bear an indirect bandgap that decreases from S to Se and then to Te. The fact of applying strains, either compressive or tensile, does not change the type of bandgap (again, except in the case of WTe_2_, which becomes indirect), but the shape of the bands can be substantially modified, potentially implying a change in the valence band minimum (VBM) and conduction band maximum (CBM); the k-points implied in the valence-to-conduction transitions are hence also changed accordingly. The bandgap evolution with respect to the applied strains is depicted in Figure 3a. It shows that, except for Te, the bandgaps in the WX_2_ bilayers are larger than in the MoX_2_ ones for a given chalcogen atom. Moreover, increasing the tensile strain leads to a bandgap shrinkage, which increases along the MTe_2_, MSe_2_, and MS_2_ sequence, irrespective of the metal atom. These tensile strain effects are similar to those observed when the materials go from a bulk state to a monolayer one: a blue shift of the energy bandgap is observed, which is attributed to quantum confinement [52]. Except for Te, for a given chalcogen atom, the shrinkage decrease is higher when the W metal is concerned. By contrast, the bandgap does not change in a systematic way as the compressive strain increases but clearly depends on the nature of both the metal atom and the chalcogen one. For WSe_2_ and MoTe_2_, the bandgap decreases when the compressive strain increases, whereas it increases for MoS_2_ and WS_2_. For the remaining compounds (MoSe_2_ and WTe_2_), the bandgap first increases and then decreases when the compressive strain increases. These results agree with those reported in the literature on bilayer MX_2_ under both uniaxial and biaxial strains [35,53]. As reported by Carrascoso et al. for monolayers, the uniaxial strains have a weaker effect on the bilayer material properties than the biaxial ones [43].

All the aforementioned effects undoubtedly have an impact on the valence-to-conduction electron transitions upon irradiation and on the excited electrons’ mobility due to band curvature changes. Indeed, the best voltage and photocurrent amplitudes are dictated by the proper balancing between the bandgap and absorbed photon energy [54]. Figure 3b plots the corresponding electron and hole effective masses (m_e_* and m_h_*, respectively) at the respective conduction and valence band edges. For each fixed X atom, MoX_2_ has a comparatively higher m* than WX_2_. Specifically, the m_e_* and m_h_* along the Γ-K direction become smaller as the tensile strain scales up, except for MoTe_2_ and WTe_2_ in the range of [0%; +2%] for which they become larger. However, in the range of [0%; −2%], the m_e_* and m_h_* along either Γ-Λ or K-Λ decrease and increase, respectively. As the compressive strain intensifies, the m_e_* and m_h_* along the K-Λ direction decrease again. It is worth mentioning that the m_e_* and m_h_* of MoTe_2_ do not follow this pattern due to the change of the electron transition path from the K-Λ to the M-Λ direction. All the above information confirms an influence of the strain on the band edges and curvatures, which can be deep enough to shift the sequence of bandgap energies and m* in MX_2_ for different X, thus providing possibilities of customizing the photovoltaic properties. Hence, considering the ideal bandgap and favorable m*, an advantageous electron excitation and transportation can be anticipated when a certain range of compressive strain is applied.

**Figure 4:**
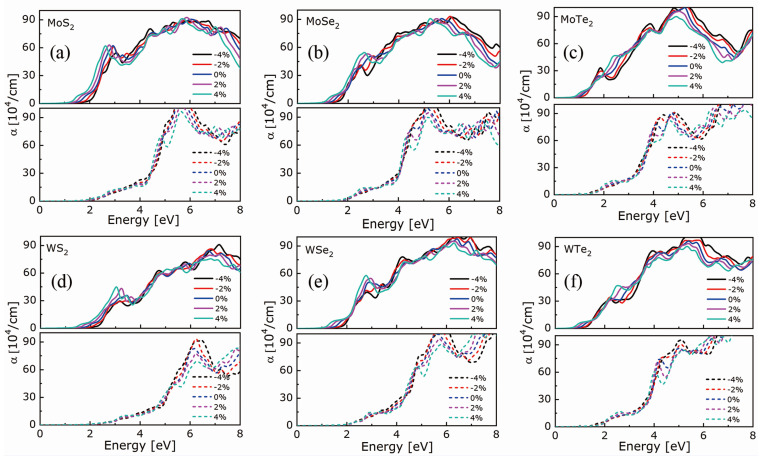
Absorption coefficients of (**a**) MoS_2_, (**b**) MoSe_2_, (**c**) MoTe_2_, (**d**) WS_2_, (**e**) WSe_2_, and (**f**) WTe_2_ in the *xx*-(top panel) and *zz*-direction (bottom panel) as the biaxial strain goes from −4% to +4%.

**Figure 5:**
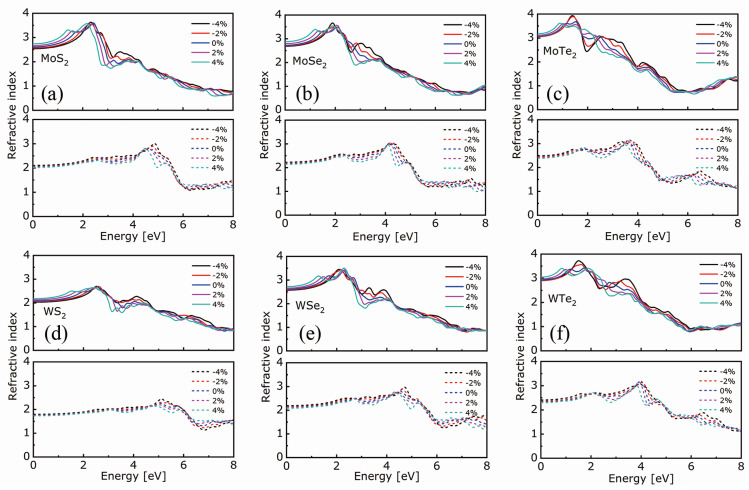
Refractive indexes of (**a**) MoS_2_, (**b**) MoSe_2_, (**c**) MoTe_2_, (**d**) WS_2_, (**e**) WSe_2_, and (**f**) WTe_2_ in the *xx*- (top panel) and *zz*-direction (bottom panel) as the biaxial strain goes from −4% to +4%.

In the following, the optical properties were analyzed in the in-plane (xx) and out-of-plane (zz) directions. The calculated absorption coefficients and refractive indexes of the investigated MX_2_ compounds are shown in Figure 4 and Figure 5, respectively. Irrespective of the compound and strain, the absorption threshold and refraction peak occur at a lower energy in the xx direction than in the zz one, with a slight, gradual shift towards higher energies from +4% to −4% strain in the case of the *xx*-direction; in the *zz*-direction, the curves are indistinguishable. Thus, for a given compound, a tensile strain allows for lowering the absorption threshold. This result agrees with the decreasing bandgap observed under tensile strain and is similar to that observed by a photoluminescence spectroscopy experiment, as the materials’ size decreases from the bulk to monolayer [3,4,55]. Irrespective of the metal atom and strain, the refraction peak intensity increases, and the absorption edge value decreases as the chalcogen atomic number increases. 

**Figure 6:**
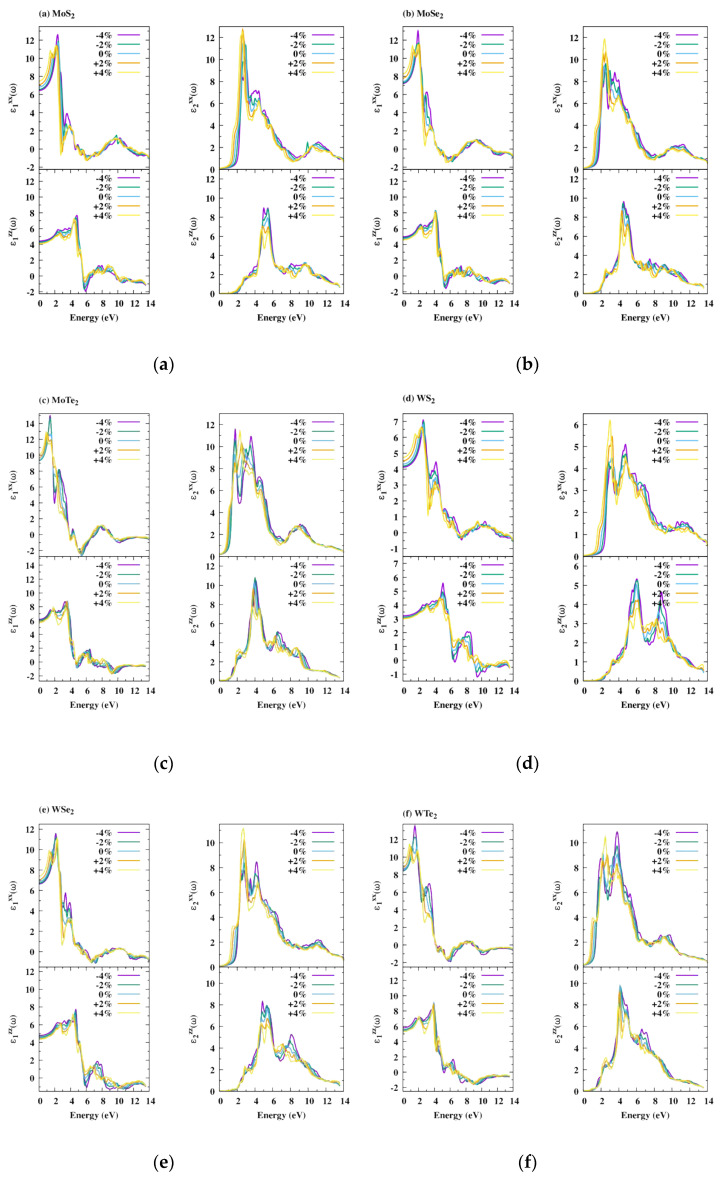
Real ε_1_ (ω) and imaginary ε_2_ (ω) parts of the relative permittivity function with respect to photon energy under biaxial, −4% and −2% compressive strains and +2% and +4% tensile ones. (**a**) MoS_2_; (**b**) MoSe_2_; (**c**) MoTe_2_; (**d**) WTe_2_; (**e**) WSe_2_; (**f**) WTe_2_.

The relative permittivity function ε (ω) is strongly related to the band structure and characterizes collective excitations close to the Fermi level [56]. The calculated dielectric functions for the six MX_2_ bilayers, subjected or not to strains, are depicted in Figure 6, with the real part ε_1_ (ω) being shown on the left panel and imaginary one ε_2_ (ω) on the right panel. For all the compounds, ε_1_ (ω) becomes negative above around 5–6 eV, which means that the compounds exhibit a metallic behavior above the photon energy thresholds [57]. At the frequency limit ω = 0, ε_1_ (0) corresponds to the static relative permittivity. For all the compounds, higher corresponding values were obtained in the *xx*-direction than in the *zz*-direction, and they increase from compressive to tensile strain, whereas they decrease in the *zz*-direction. Irrespective of the metal atom, ε_1_ (0) increases with the chalcogen atomic number. Hence, based on the Penn model [58], which defines ε_1_ (0) as $\epsilon_{1}\left(0\right)\approx 1+{\left(\frac{\hbar \omega }{E_{g}}\right)}^{2}$, the bandgap should decrease from S to Te, which is indeed observed in Figure 3a. The highest static relative permittivity value was obtained for MoTe_2_, indicating a higher polarizability for this bilayer. These values are further improved in the *xx*- and *zz*-direction when the compound undergoes tensile and compressive strains, respectively. For all the compounds, when compared with ε_2_ (ω) in the *xx*-direction, we observe that ε_2_ (ω) in the *zz*-direction tends to decrease and that its peak maximum shifts towards higher incident photon energies. These results indicate a decrease in the ability of the compounds to absorb light in this direction.

### 3.2. Electron Density Analysis

In the realm of the quantum theory of atoms in molecules (QTAIM) [29,50] the key fields are the electron density, and especially its Laplacian, from which numerous parameters were derived, which enables the characterization of the bonding between atoms in molecules and crystals [59]. Among these parameters, the bond degree (BD) at the bond critical point BD = H_b_/ρ_b_, where H is the total energy density, i.e., the sum of the potential V and kinetic G energy densities, and ρ is the electron density at the bond critical point (b), measures the degree of covalence (BD < 0) or softening (BD > 0) of the interatomic bonding [51]. In other words, covalent bonds are characterized by large, negative values of BD, whereas closed-shell interactions (ionic and van der Waals interactions) are characterized by positive BD values.

**Figure 7:**
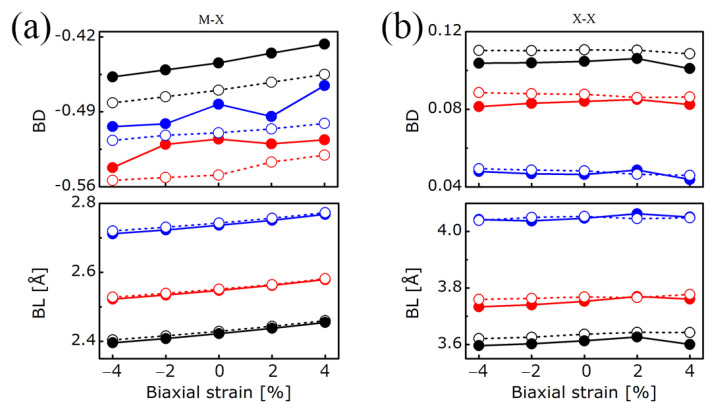
Bond degrees (BD) and bond lengths (BL) of (**a**) M-X and (**b**) X-X bonds in MX_2_ bilayers (M = Mo, W; X = S, Se, Te) vs. biaxial strain. Mo: solid circle; W: hollow circle; S: black line; Se: red line; Te: blue line.

**Figure 8:**
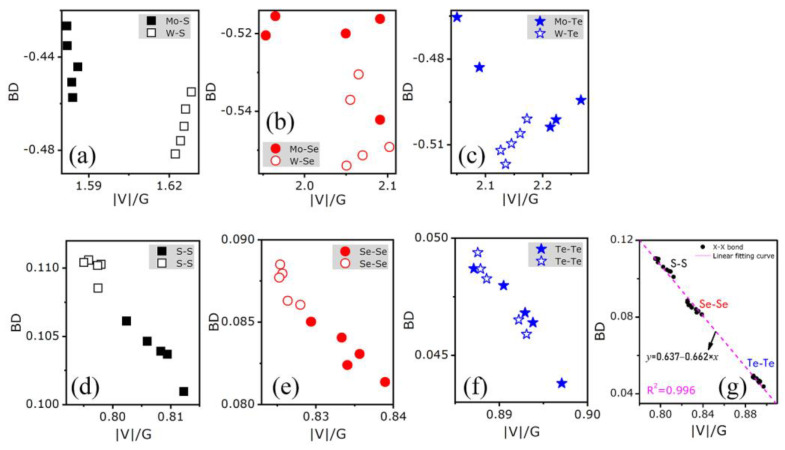
Bond degrees (BD) vs. |V|/G of M-X bonds in (**a**) MS_2_, (**b**) MSe_2_, (**c**) MTe_2_, and X-X bonds in (**d**) MS_2_, (**e**) MSe_2_, and (**f**) MTe_2_. Full symbol: Mo-containing bilayers; hollow symbols: W-containing bilayers. (**g**) Linear fitting result of BD vs. |V|/G of X-X bonds.

Figure 7 and Figure 8 depict the bond degree of both the M-X and X-X bonds for the bilayers subjected to strains. In addition, the evolution of the M-X and X-X bond lengths under strains are also depicted in Figure 7. Unsurprisingly, for both the M-X and X-X bonds, the BL increases with the chalcogen atomic number, irrespective of the metal atom. For a given chalcogen atom, the Mo-X and W-X bond lengths are the same for each applied strain, which can be explained by the similar value of the Mo and W covalent radii (145 pm and 146 pm, respectively). The M-X bond lengths linearly increase when the strain varies from −4% to +4%. In the case of the X-X bond lengths, a slight difference is noticeable depending on whether Mo or W is bonded to the chalcogen atom. This slight difference is also observed in the corresponding bond degrees, which, contrary to the bond lengths, decrease when the chalcogen atomic number increases. This decrease reflects the lowering of the van der Waals character of the interatomic interaction. Regarding the evolution with the strain, both the X-X bond lengths and bond degrees are nearly constant. For the M-X bonds, the BDs increase from compressive to tensile strains, the absolute values of which are increasingly large for S, Te, and Se.

According to Figure 8, there is no clear relation between BD and |V|/G values of M-X bonds. By contrast, a linear relationship can be evidenced for the X-X ones, the slope being the same for all the compounds (see Figure 8g) with or without strain. However, irrespective of the bond and the compound, the evolution of both BD and |V|/G values with respect to strain seems to be weak. In order to better evaluate this evolution, the G/ρ value, which corresponds to the slope of the line passing through the point of interest and that of the coordinates |V|/G = 1 and BD = 0 (see [60]), was determined for each bond type in each bilayer with and without strains. The results are gathered in Table 1. One can see the following: (i) the G/ρ values for each bond are almost independent of strain; (ii) the G/ρ values of both X-X and M-X bonds mainly depend on the X atom, the influence of the M one being very weak; (iii) the G/ρ values decrease when the atomic number of the X atom implied in the bond increases; (iv) for X= Se and Te, the G/ρ values of M-X and X-X bonds are very close to each other, and for X = S, the G/ρ values of M-X bonds are higher than those of the X-X ones. These results agree with the inverse relation between the kinetic energies per electron G/ρ at the bond critical point and the bond polarizability, as proposed by Yang et al. [60]. Indeed, the larger chemical softness of Te compared to that of Se and S, and the larger one of Mo compared to that of W, should correspond to larger polarizability. This can be related to the highest polarizability evidenced in the previous section for MoTe_2_ from static relative permittivity values.

**Figure 9:**
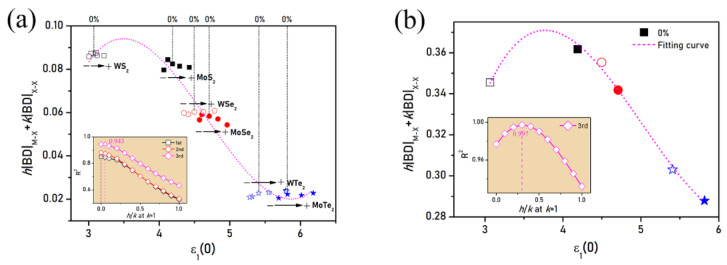
Results of h|BD|_M-X_+k|BD|_X-X_ vs. ε_1_ (0) of the MX_2_ bilayer structures (**a**) under biaxial strain range of [−4%; +4%] and (**b**) under no strain at their maximal fitting coefficient of determination R^2^. Inset in (**a**) depicts the first-, second- and third-order fittings of R^2^ vs. h/k. Inset in (**b**) depicts the third-order fitting of R^2^ vs. h/k ratio. Arrows in between symbols “−” and “+” in (a) are used to represent the evolution from the biaxial compressive (−4%, −2%) strains to the tensile (+2%, +4%) ones.

According to Gatti [59], an atomic expectation value results from the sum of bond contributions. As we did in previous works [28,61], a relationship was searched for between the bond degrees summation and the relative permittivity under zero frequency ε_1_ (0) along the *zz*-direction. As two types of bonds coexist in the structures, namely M-X and X-X, the summation can be written as h|BD|_M-X_+k|BD|_X-X_, with h and k the parameters to be fitted. Fitting this expression via the equation, the maximum coefficient of determination R^2^ is obtained by adjusting h and k at each equation order. Irrespective of the strain, the most accurate description of the relationship between bond degree and static relative permittivity is given by a cubic equation (see Figure 9a), namely, *n* = 3. The absolute BD summation and the ε_1_ (0) are inversely related. The best fit at *n* = 3, for which R^2^ = 0.945, was obtained for h/k = 0.05, in comparison to the best fit at *n* = 1 and *n* = 2, for which R^2^ = 0.849 and R^2^ = 0.881, respectively, both obtained for h/k = 0.00. These results indicate that in strain-modified MX_2_ bilayers, the X-X bonds are overwhelmingly contributing to the dielectric properties. By contrast, the fitting result under no strain, as seen in Figure 9b, shows a perfect cubic relationship between the bond degree summation and static relative permittivity with the R^2^ = 0.997 for h/k = 0.3. This is coherent with our previous observation in the absence of strains [28], where both types of bonds were found to participate in achieving this cubic relationship, although the X-X bonds were found to contribute more than the M-X ones. The profound decrease of h/k ratio of MX_2_ from no applied strain to added strain highlights the pronouncing role of the X-X bonds in responding to the external photoelectric field, as proven by the manifest dependence of the absorption and refractive properties on the strain as X varies. 

## 4. Conclusions

The influence of biaxial strain on optoelectronic properties and electron density of transition metal dichalcogenides MX_2_ (M = Mo, W and X = S, Se, Te) bilayers were thoroughly investigated for the first time using DFT calculations. When subjected to a strain going from a compressive (−4%, −2%) to tensile (+2%, +4%) one, the 2D materials’ band structures and their corresponding effective masses of charge carriers undergo non-symmetrical changes as compression and tension are concerned. The bandgap shrinks remarkably as tensile strain increases, concomitantly with both electron and hole effective masses lowering, except for those of MoTe_2_. By contrast, when a compressive strain is applied, the bandgap and electron effective masses evolve at a much slower rate. In the meantime, the hole effective masses first increase and then decrease as strain goes from 0% to −4%. Nevertheless, the strain-induced bandgap shrinkage shall be strong enough to alter the strain-free bandgap energy sequence following MTe_2_, MSe_2_, MS_2_. Irrespective of the compound and strain, the absorption threshold and refraction peak occur at a lower energy in the in-plane (xx) direction than in the out-of-plane (zz) one. A strain effect is only visible in the *xx*-direction. For a given compound, the absorption threshold is lowered when subjected to a tensile strain. Irrespective of the metal atom and strain, the refraction peak intensity increases, and the absorption edge value decreases as the chalcogen atomic number increases. The strain effect on absorption and refraction is direction dependent. More precisely, these values are further improved in the *xx*- and *zz*-direction when the compound undergoes tensile and compressive strains, respectively. From the determination of the bond degree (BD) at the bond critical points using QTAIM, it was found that the X-X BD decreases when the chalcogen atomic number increases and is nearly constant with applied strains. The kinetic energy per electron G/ρ at the bond critical point was also estimated. It is almost independent of strains with the lowest values for X = Te, which can be related to the highest polarizability evidenced in MoTe_2_ from static relative permittivity values. A cubic relationship between the absolute BD summation of M-X and X-X bonds and the static relative permittivity was observed both in strained and unstrained MX_2_ bilayers. After applying strain, the preponderant contribution of the X-X bonds in this relation under no strain was substantially reinforced, yielding the leading role to the X-X bonds instead of the M-X ones in the photovoltaic response. As the application of a biaxial strain on the chalcogenides bilayers allows for reproducing the effect of epitaxial strain on the absorber layer in a real device, these results can be valuable for the building of photovoltaic devices.

## Figures and Tables

**Table 1:** G/ρ values of M-X and X-X bonds in strained and unstrained MX2 (M = Mo, W; X = S, Se, Te) bilayers.

Strain (%)	MoS2		MoSe2		MoTe2		WS2		WSe2		WTe2	
	Mo-S	S-S	Mo-Se	Se-Se	Mo-Te	Te-Te	W-S	S-S	W-Se	Se-Se	W-Te	Te-Te
−4	0.78	0.54	0.50	0.50	0.42	0.44	0.77	0.55	0.53	0.5	0.46	0.44
−2	0.77	0.54	0.55	0.50	0.41	0.44	0.76	0.54	0.52	0.5	0.45	0.43
0	0.76	0.54	0.53	0.50	0.44	0.44	0.75	0.54	0.50	0.5	0.45	0.43
+2	0.75	0.54	0.50	0.50	0.39	0.43	0.74	0.54	0.51	0.5	0.44	0.43
+4	0.73	0.54	0.47	0.50	0.44	0.43	0.72	0.54	0.50	0.5	0.43	0.43
